# The protective role of TBX21-1514T>C polymorphism in susceptibility to multiple sclerosis

**Published:** 2018-07-06

**Authors:** Fatemeh Akbarian, Mitra Ataei, Zivar Salehi, Masoud Nabavi, Mohammad Hossein Sanati

**Affiliations:** 1 Department of Medical Genetics, National Institute of Genetic Engineering and Biotechnology, Tehran, Iran; 2 Department of Biology, School of Basic Sciences, University of Guilan, Rasht, Iran; 3 Department of Neurology and Neuroregenerative, Royan Institute, Tehran, Iran

**Keywords:** Multiple Sclerosis, Genetic Polymorphism, T-bet Transcription Factor Gene, Interferon-Gamma

## Abstract

**Background:** As a T-cell mediated disease, multiple sclerosis (MS) pathogenesis might be associated with the immune system and its involved genes. TBX21, which encodes T-bet transcription factor, is a critical regulator of the commitment to the Th1 lineage and Interferon gamma (IFNγ) production. Investigation of the association of -1514T > C polymorphism located upstream of TBX21 gene with MS susceptibility is reasonable due to its demonstrated significant association with some other immune-mediated diseases.

**Methods:** We analyzed the genotype frequencies of -1514T > C polymorphism between 248 Iranian patients with MS and 163 matched healthy controls. By applying polymerase chain reaction-single strand conformation polymorphism (PCR-SSCP)- technique, the single-strand conformation patterns of the amplicons were compared and sequenced.

**Results:** Strong association between the wild -1514T allele and MS susceptibility was found with the allelic frequency of 99.6% in patients vs. 95.1% in controls (P = 0.002), and the CC genotype frequency of the TBX21 polymorphism (-1514T > C) reported potential protective effect against the disease (P = 0.014).

**Conclusion:** The TBX21-1514T > C polymorphism confers possible protective effect on MS in Iranian population. Further comprehensive studies in different settings are required to clarify the exact role of TBX21 gene in MS disease.

## Introduction

Multiple Sclerosis (MS) is an immunologically associated disorder of the central nervous system (CNS), being characterized by demyelination and degeneration of neural cells.^   1 ^ Being considered as a multifactorial disease, genetic plays a major role in MS.^   2 ^ The first and most significant genetic association was identified with major histocompatibility complex (MHC) alleles in 1972;^   3 ^ where many of the related genes are involved in various immunological processes. Moreover, several studies in the past 30 years have demonstrated a common association of HLA-DRBI*1501 allele with genetic risk for MS in different societies.^   4 ^ Despite recent findings, the complexity of this disease has convinced scientists to follow more genetic clues. Thus, recognition of pathological factors and their involved genes would possibly affect the route to more identification.

The pathogenesis of MS is mediated by the development of auto-aggressive T-lymphocytes in peripheral immune organs, which migrate through the blood-brain barrier (BBB); thus, triggering inflammation, and eventually leading to demyelination and degeneration of axons.^     5 ^ The developmental regulation of auto-reactive T-helper cells is mainly determined by various transcription factors such as T-bet, which involves with up-regulating the production of interferon gamma (IFNγ) in Th1 cell subtypes.^   6 ^ Considering the autonomous activity of Th1 cells as one reason for MS, many evaluations have been done on T-bet transcription factor. As reported by Fazeli, et al.,^   7 ^ increased expression of T-bet is demonstrated in the animal model contributed to MS disease, known as experimental autoimmune encephalomyelitis (EAE). Interestingly, T-bet-deficient mice are protected from EAE progression,^        8 ^ and T-bet silencing in vivo has shown inhibiting effects on EAE development;^        9 ^ thus confirming the necessity of T-bet encoding gene, TBX21, in MS pathogenesis. Although recent evaluations confer a major role for Th17 cell cytokines and transcription factors in MS,^   10 ^ T-bet is still necessary for the survival of Th17 cells through regulating the expression of the IL-23 receptor.^        11 ^ Furthermore, many studies indicate a boost in TBX21 expression during relapses of patients with Relapsing-Remitting MS (RRMS),^   12 ^ suggesting it as a probable biomarker in immune-related diseases.^   13 ^

Despite the importance noted for TBX21, no study has yet been conducted on the molecular polymorphisms of this gene in patients with MS. We therefore set forth to investigate the potential presence of single nucleotide polymorphisms (SNP) in TBX21 gene in susceptibility to MS among the Iranian patients. According to several case-control studies, various SNPs have shown association with other immune-related diseases, especially in the promoter region upstream of this gene.^14^^-^^16^ Between upstream promoter variants, -1993T>C and -1514T>C polymorphism were the most significant in association with systemic lupus erythematosus (SLE) and rheumatoid arthritis (RA).^14^^,^^17^ Consequently, there seems to exist a possible relationship between TBX21 gene promoter region SNPs and MS disease, which has not been inspected so far. Thus, in this study, we hypothesized an association between TBX21 promoter polymorphisms with MS susceptibility.

## Materials and Methods

***Patients and deoxyribonucleic acid (DNA) samples:*** Blood samples were collected from 248 definite MS patients, initially diagnosed by neurologist according to the McDonald criteria.^     18 ^ All of the patients were between 18-55 years old (mean age of 34 ± 2), 78% originally from north and west of Iran, with the expanded disability status scale (EDSS) of 0-6 (mean: 3), and mean disease duration of 4.00 ± 0.07 years, with female/male ratio of about 2 folds. Moreover, 163 healthy controls without any clinical symptoms or familial history of MS or other autoimmune disorders were also matched by age, gender, and place of birth (Table 1). The study was approved by the Ethical Committee of National Institute of Genetic Engineering and Biotechnology, Tehran, Iran, (ethical code: IR.NIGEB.EC.1395.4.1.B), and informed written consent was obtained for all the MS patients and healthy controls. Genomic DNA was then extracted from the leukocytes of the peripheral blood by using a genomic DNA extraction kit produced by Molecular Biological System Transfer (MBST) Institute, Tehran City, based on the manufacturers’ protocol.

***Mutation screening: ***All the DNA samples were analyzed using single strand conformation polymorphism (SSCP) and DNA sequencing procedure. 

**Table 1:** Demographic and clinical characteristics of patients with multiple sclerosis (MS) and controls

**Demographic data**	**Healthy controls (n = 163)**	**Patients with MS (n = 248)**
Mean age (year) (range)	37.9 (21-63)	34 (18-55)
Sex (female/male ratio)	2.05	1.93*
Mean EDSS with 0-10 points (range)	n/a	3 (0-6)
Mean disease duration (year) (range)	n/a	4 (0.08-17.00)
MS-type (n)	n/a	RRMS (195)

Not applicable

MS: Multiple sclerosis; EDSS: Expanded disability status scale; RRMS: Relapsing-remitting MS

* This value is referred to the Sex (female/ male ratio) in patients with MS. It means that the female population with MS is approximately 2 folds to the male population with this disease.

Concisely, particular primer pairs were designed for the 5’ flanking promoter region of TBX21 gene (5’- GTGAAGGTAGAGAGAGGAGAAG- 3’ and 5’- CAGCACAGAAAAGTAAAAACAAGA- 3’ as forward and reverse primer, respectively) in order to replicate the extent of 333 bp for each amplicon. Polymerase chain reaction (PCR) amplification technique was performed in a final volume concentration of 25µl and under standard reaction conditions.^   19 ^ For the SSCP analysis, PCR-amplified segments were denatured and separated in 10% polyacrylamide (39:1) in 5x Tris-Borate-EDTA (TBE) gels, and were run at 90-105 W, for 12 hours and at room temperature of 4 °C. Further details are presented upon request.

The PCR amplicons with diverse electrophoretic migration patterns through SSCP technique were resolved by direct sequencing (ABI 3730XL Analyzer, Applied Biosystems, CA, USA), trace files were checked and edited using FinchTV1.4.0. (Geospiza Inc., Seattle, USA). Sequences were aligned and evaluated manually using Clustal X 2.0.11 software.

In this report, the chi-square test was used to examine the differences between various genotypes and allelic frequencies between patients and healthy controls. Furthermore, the logistic regression, odds ratio (OR), and 95% confidence interval (CI) were applied to determine the relationship between the genotypic and allelic frequencies with MS disease. Statistical analysis was performed using SPSS software (version 13, SPSS Inc., Chicago, IL, USA). A P-value of less than 0.05 was considered to specify statistical significance.

## Results

**Figure 1:**
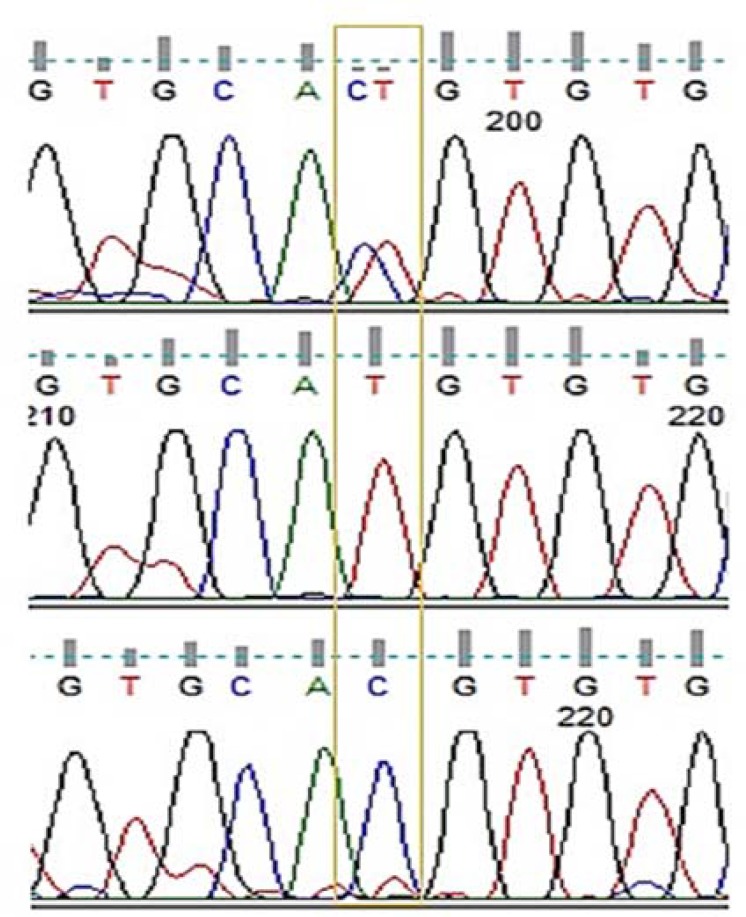
Electropherograms representing -1514T>C polymorphism in the promoter region of TBX21 gene. The box indicates the position of the single nucleotide polymorphisms (SNP) according to NG_012166.1. The three genotypes of CT heterozygote, TT homozygote, and CC homozygote are demonstrated from top to bottom, respectively.

In order to evaluate the association of TBX21 gene with MS disease, a mutational analysis was conducted using PCR-SSCP technique. Accordingly, 3 different electrophoretic patterns were obtained for the amplicons in both patients and healthy controls; however, an unambiguous localization of the mutation site was not allowed. Thus, the 333 bp amplified promoter region was submitted to DNA sequencing analysis, where the alignments clearly indicated the presence of a SNP noted in the -1514T>C (rs17250932). The electropherogram revealed 3 genotypes for -1514T>C in the promoter region (Figure 1), interestingly coinciding with the migration patterns obtained by SSCP electrophoretic bands.

Moreover, the allelic and genotypic frequencies were analyzed and the results were presented as P-value, OR, and 95% CI in table 2.

**Table 2:** The odds ratio (OR) and P-value of -1514T>C in the promoter region of TBX21 gene

**SNP**	**Cases [n (%)]**	**Controls [n (%)]**		**OR**	**CI (95%)**	**P**
Genotype (χ^2^ = 9.760, df = 2)						0.008
CT	68 (27.4)	38 (23.3)		1*	-	-
CC	1 (0.4)	8 (4.9)		14.320	1.730-118.840	0.014
TT	179 (72.2)	117 (71.8)		1.170	0.740-1.850	0.505
Allele (χ^2^ = 9.319, df = 1)						0.002
C	1 (0.4)	8 (4.9)		1**	-	-
T	247 (99.6)	155 (95.1)		0.078	0.010-0.633	0.017

SNP: Single nucleotide polymorphisms; OR: Odds ratio; CI: Confidence interval; df: Degree of freedom

* The reference category is: CT,

** The reference category is: C

The findings presented in table 2 showed that among patients with MS, 27.4% had CT genotype, 0.4% had CC genotype, and 72.2% were with a TT genotype. Also among the healthy controls, 23.3% had CT genotype, 4.9% had CC genotype, and 71.8% were with a TT genotype. Chi-square obtained (χ^2^ = 9.760) with 2 degrees of freedom (df) is significant with P = 0.008; and the existence of a significant relationship will thus be concluded between the genotype and the risk of MS in the population. Additionally, the logistic regression supports the significant association of CC genotype with reduced MS disease (OR = 14.32, 95% CI = 1.73-118.84, P = 0.014).

Moreover, 0.4% of the MS patients had the allelic frequency of C, while the rest of 99.6% of them had a T allele. In healthy controls, this frequency was shifted to 4.9% and 95.1% for C and T alleles, respectively. Chi-square obtained (χ^2^ = 9.319) with df = 1 is significant with P = 0.002, meaning that there is a significant association between the allelic frequency and MS disease. Thus, genotypic and allelic frequency of -1514T>C polymorphism in MS patients is significantly different from the healthy controls (P < 0.050).

## Discussion

Genetic and environmental factors take part in the initiation and progression of MS.^   20 ^ In the recent genome-wide association studies (GWAS), many loci have been identified with great impact on the disease;^        21 ^ where some reports suggest presence of heavily fortified areas of immunological genes.^        22 ^ According to the combined results from GWAS and the internationally collaborative ImmunoChip experiment,^21^^,^^23^ the role of the immune system in MS is mostly supported by the overlap of roughly half of its risk genes with other immunological diseases, such as celiac disease, Crohn's disease, primary biliary cirrhosis, type I diabetes, and RA.^        24 ^ These immune-related genes are generally located in the regulatory regions of DNA sequences, such as introns, promoters, and intragenic regions.^        25 ^ Therefore, it indicates that MS susceptibility is regulated through altered immune cell differentiations in leukocytes, with transcription factors and cytokines as the master regulators.

Among T-lymphocytes, great attention has controversially been paid to Th1 and Th17 cells in MS disease, since taking the major part in the induction of EAE model through the production of IFNγ and IL-17 cytokines, respectively.^26^^,^^27^ Yet, among Retinoic acid receptor-related orphan receptor gamma (RORγt) and T-bet as the transcription factors associated with Th17 and Th1, respectively, T-bet remains to be essential for the pathogenic nature of auto-reactive T-cells.^        9 ^ Evaluating T-cell contents in the CNS commonly reveals strong production of IFNγ and expression of the Th1-master regulator T-bet.^   6 ^ Moreover, Th17 cells can convert to T-bet positive during inflammation in order to maintain their pathogenic potentials, due to their plasticity.^        28 ^

In addition to the evidence obtained from the EAE models, several studies suggest an association between T-bet and relapses in MS and comparable autoimmune diseases, such as asthma, SLE, and systemic sclerosis.^12^^,^^29^^,^^30^ Furthermore, a reduction was shown in the expression level of T-bet in peripheral blood of improved patients with one year of IFN-В treatment.^   31 ^ These reports make TBX21 a potential candidate involved in MS development. 

Based on the research conducted on TBX21 promoter region, multiple up- and down-regulatory sequences have been found 2 kb upstream of transcription start site,^        32 ^ suggesting an enriched polymorphic region. These polymorphisms, including two significant -1993T>C and -1514T>C SNPs, have had shared effects on several immune-related diseases such as hepatitis type I, SLE, RA, and systemic sclerosis.^15^^,^^17^^,^^33^ It should be noted, however, that a similar study has not been performed on MS disease so far. 

In this research, we have investigated the genotypic and allelic frequencies at -1514T>C SNP of the TBX21 gene between the MS patients and normal controls. When analyzing, no association with MS could be demonstrated; however, a significant protective effect was discovered in MS subgroups (P = 0.008). As a result, -1514CC genotype was found to be strongly protective, reducing the odds of MS (OR = 14.32, 95% CI: 1.73-118.84, P = 0.014). These results were confirmed by the putative function, recently reported for this SNP in lupus erythematous.^        14 ^ The upstream promoter region of TBX21 functions as an E-box-binding site, which has complementary region with -1514C allele. The E-box family transcription factors, such as upstream stimulatory factor (USF)-1 expressed in peripheral T-cells, are considered as key regulators of the genes involved in immune responses and cell growth.^        34 ^ The USF-1 transcription factor directly or indirectly regulates the expression of cytokines and MHC genes.^        35 ^ Accordingly, -1514C allele influences the expression level of TBX21 by mediating the optimal binding affinity between USF-1 and the promoter region.^        14 ^ In the same survey conducted, it was shown that lower levels of IFNγ are produced by CD4+ Th1 cells with -1514CT heterozygote allele compared with -1514TT homozygote allele.^        14^

Interestingly, our data indicates that the -1514T allele is significantly associated with increased MS disease (OR = 0.078, 95% CI: 0.010-0.633, P = 0.017), possibly because of reducing the binding affinity of USF-1 to the promoter region; and thus, it might allow over expression of T-bet transcription factor, leading to the up-regulation of IFNγ and auto-reactivity of Th1 cells, respectively. As auto-aggressiveness of T-cells is a hallmark of MS disease pathogenicity, it is likely that this function of TBX21 might play a great role in this orchestrate, and thus is worthy of further investigation.

## Conclusion

It was not improbable to predict the presence of important TBX21 polymorphisms in MS, considering the common features between the diseases termed earlier and MS concerning their immune-mediated pathogenesis. On the other hand, by skimming through various resources and reports associated with the MS disease, no investigation has been reported on the promoter of TBX21 gene polymorphisms up to date, and this represents an innovation in this research. This novel genetic association will be important for future studies that will determine whether or not this polymorphism takes part in the pathogenesis of MS disease; where further epidemiological and functional studies are required. In summary, our results suggest a potential protective role for -1514T>C polymorphism in MS pathogenesis.

## Notes:

**How to cite this article:** Akbarian F, Ataei M, Salehi Z, Nabavi M, Sanati MH. The protective role of TBX21-1514T>C polymorphism in susceptibility to multiple sclerosis. Iran J Neurol 2017; 17(3): 111-6.
